# Epidermoid Causing Ischemic Stroke in the Brainstem

**DOI:** 10.1155/2014/801615

**Published:** 2014-12-15

**Authors:** Raghvendra Ramdasi, Amit Mahore, Abhijeet Kulkarni, Vithal Rangarajan, Manoj Patil, Juhi Kawale

**Affiliations:** ^1^Department of Neurosurgery, King Edward Memorial Hospital and Seth Gordhandas Sunderdas Medical College, Parel, Mumbai 400012, India; ^2^Department of Medicine, King Edward Memorial Hospital and Seth Gordhandas Sunderdas Medical College, Parel, Mumbai 400012, India

## Abstract

Intracranial tumors may rarely cause stroke. We report an epidermoid cyst causing stroke in a pediatric patient. We have also reviewed the literature and pathogenesis of stroke caused by intracranial tumors.

## 1. Introduction

Stroke as a presenting symptom of intracranial tumors is extremely rare. Epidermoids referred to as “an ordered disorder” usually produce symptoms of slowly progressive mass lesion [1]. We report a pediatric case of brainstem infarct caused by this “well mannered” epidermoid tumor in cerebellopontine angle. This phenomenon was only once reported in adults [2].

## 2. Case Presentation

**Figure 1:**
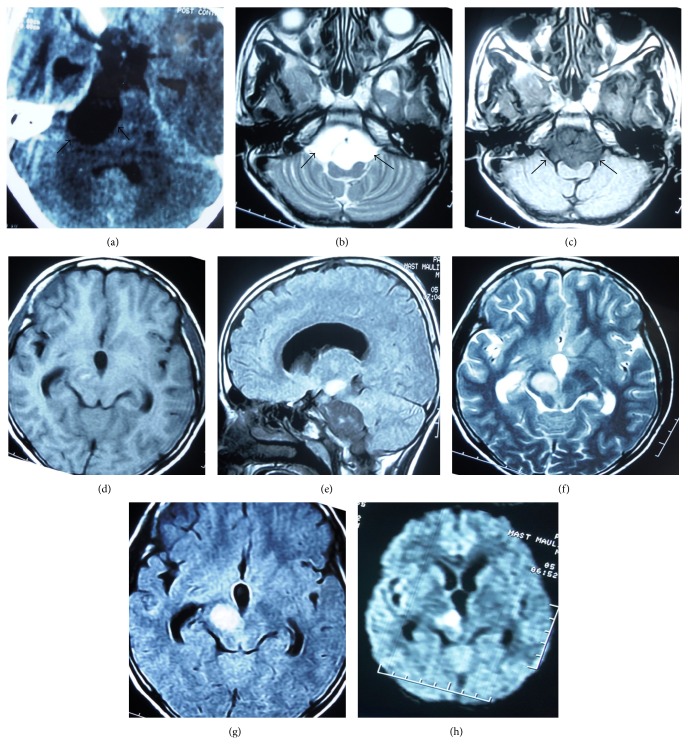
(a) Axial image of plain computed tomography (CT) showing hypodense lesion in right cerebellopontine angle and prepontine cistern with compression of the brainstem. Axial T2 (b) and T1 (c) weighted MR images showing the lesion to be hypointense on T1 and hyperintense on T2 sequence (black arrows outline the tumour). (d) and (e) are T1 weighted axial and sagittal images. (f), (g), and (h) are axial T2, FLAIR, and diffusion weighted images, respectively, revealing another lesion of hyperintensity and restricted diffusion in right half of midbrain.

A 12-year-old boy was brought by parents into a rural hospital with complaint of headache for the last 6 months. Computed tomography (CT) scan of brain revealed a hypodense lesion in right cerebellopontine angle and basal cistern (Figure 1(a)). The provisional diagnoses were arachnoid cyst and epidermoid cyst. The patient was referred to tertiary care centre for further management as neurosurgical and magnetic resonance imaging (MRI) facilities were not available at the rural hospital. However, the parents did not follow the medical advice. He was brought to our emergency department after 5 months when he developed altered sensorium and left hemiparesis of sudden onset. On examination, the patient was drowsy and not obeying commands. The patient had good spontaneous activity of right sided limbs without any spontaneous movements of left sided limbs. There was definite paucity of movements of left sided limbs to the central painful stimuli as compared to those of the right sided limbs. The exact assessment of weakness was not possible due to the drowsy state. The right pupil was larger as compared to the left pupil and was not reacting to light. The left sided limbs had hyperreflexia. The left plantar reflex showed extensor response. The rest of the findings were unremarkable.

Magnetic resonance imaging (MRI) of the brain was done within one hour of arrival of the patient and approximately 12 hours after the onset of symptoms. It revealed the lesion with isointensity on T1 weighted imaging and hyperintensity on T2 weighted imaging in right cerebellopontine angle extending to basal cistern (Figures 1(b) and 1(c)). There was another lesion in right half of midbrain with hyperintensity on T1, T2, and FLAIR images (Figures 1(d), 1(e), 1(f), and 1(g)), restricting diffusion on diffusion weighted image (DWI) like the first lesion (Figure 1(h)). The lesion showed low signal on apparent diffusion coefficient (ADC) map ruling out T2 shine-through effect. These findings were suggestive of right cerebellopontine angle epidermoid with right midbrain infarct. The restriction of diffusion on DWI ruled out the possibility of arachnoid cyst. His other investigations including coagulation profile and electrolytes were normal.

The patient was operated on, on emergency basis via right retrosigmoid approach, and near-complete excision of epidermoid cyst was done leaving behind the capsular fragments densely adherent to vessels and nerves.

**Figure 2:**
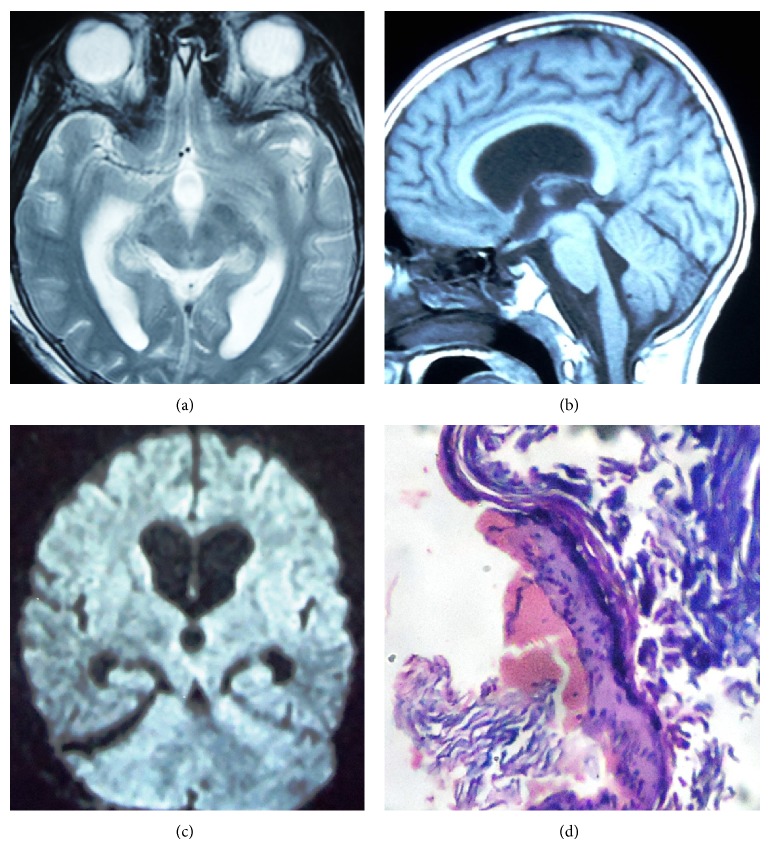
(a) Axial T2, sagittal T1 (b), and diffusion weighted postoperative image (c) showing complete excision of the lesion and resolution of the infarct. (d) The photomicrograph of the lesion (H&E, 100x) showing stratified squamous lining epithelium with lamellated keratin.

The patient improved in sensorium with partial improvement of hemiparesis after the surgery. Magnetic resonance imaging (MRI), done one month after the surgery, was suggestive of complete resolution of the infarct (Figures 2(a), 2(b), and 2(c)). Histopathological examination revealed the lesion lined by stratified squamous epithelium with lamellated keratinconsistent with epidermoid cyst (Figure 2(d)). At follow-up of 1 year after the surgery he was completely normal.

## 3. Discussion

Schnitker and Lehnert in 1952 described middle cerebral artery infarct in a case of pituitary apoplexy [3]. There are few cases of intracranial tumors causing infarct, the mechanisms of which differ from each other [2]. Pituitary adenoma is the most common benign tumor causing infarct. These encase the vessels and compress them subsequently. Infrequently these may cause vasospasm [2, 4]. Meningiomas cause infarct by compression of the vessels [5]. Malignant gliomas cause infarct by invasion of vessels [6–8]. Ruptured dermoid cyst results in vasospasm and infarct [9, 10]. One case of ruptured craniopharyngeal cyst causing infarct is reported and vasospasm is the implicated mechanism [11].

Epidermoids account for approximately 1% of all intracranial tumors. Although these lesions are congenital, patients are usually symptomatic in their third to fifth decade [12]. Two cases of epidermoid causing brainstem infarct were reported where stretching of the vessel was the postulated mechanism [2]. It is likely that the tumor in our case stretched one or more branches of the basilar artery leading to the infarct.

In all the cases reviewed, we found only one pediatric case described by Pozzati et al. [13]. Our case is the second paediatric case of tumors causing infarct and the youngest one. It is the first pediatric case and the third case of epidermoid causing infarct.

T1 hyperintensity with no blooming on gradient echo as in our case may represent incomplete stroke as described by Baheti et al. and warrant urgent intervention [14].

The two cases described before were treated medically for two weeks and the postoperative improvement in their deficits is not mentioned [2]. In our case we operated on the patient within 16 hours of the ictus and he improved remarkably in his deficits. We did not give antiplatelet treatment to our patient because surgery was anticipated in our case. The use of antiplatelets is known to increase the incidence of hematoma in major neurosurgical procedures.

## 4. Conclusion

Epidermoids may cause acute symptoms as brainstem infarct. The exact mechanism is unknown, but the stretching of the vessels may play an important role. Early surgery instead of medical treatment can reverse the insult. When primary cause of stroke is tumor or tumor-like lesion, the early surgery rather than antiplatelet treatment should be considered.
